# The added value of the EQ-5D with a cognition dimension in injury patients with and without traumatic brain injury

**DOI:** 10.1007/s11136-019-02144-6

**Published:** 2019-02-28

**Authors:** A. J. L. M. Geraerds, Gouke J. Bonsel, Mathieu F. Janssen, M. A. de Jongh, Inge Spronk, Suzanne Polinder, Juanita A. Haagsma

**Affiliations:** 1 Department of Public Health, Erasmus MC, University Medical Center Rotterdam, P.O. Box 2040, 3000 CA Rotterdam, The Netherlands; 2 Section Medical Psychology and Psychotherapy, Department of Psychiatry, Erasmus MC, Rotterdam, The Netherlands; 3 Division Mother and Child, Utrecht University Medical Center, Utrecht, The Netherlands; 4 Department Trauma TopCare, ETZ Hospital, Hilvarenbeekseweg 60, 5022 GC Tilburg, The Netherlands

**Keywords:** HRQoL, EQ-5D, Cognition, TBI

## Abstract

**Purpose:**

This study investigated the psychometric gain, if any, from the extension of the EQ-5D with a cognition bolt-on (EQ-5D + C) in a large cohort injury patients with and without traumatic brain injury (TBI).

**Methods:**

Hospitalized adult injury patients filled out a survey 1 month after initial admission. The survey included the EQ-5D-3L, the cognition bolt-on item in EQ-5D format, and the visual analogue scale (EQ-VAS). We compared ceiling and other distributional effects between EQ-5D and EQ-5D + C and TBI and non-TBI group, and assessed convergent validity using the predictive association with EQ-VAS. Also, we assessed explanatory power using regression analysis, and classification efficiency using Shannon indices.

**Results:**

In total, 715 TBI patients and 1978 non-TBI patients filled out the EQ-5D + C and EQ-VAS. Perfect health was reported by 7.9% (*N* = 214) on the EQ-5D, and 7.3% (*N* = 197) on the EQ-5D + C. Convergent validity was highest for EQ-5D + C in the TBI group (Spearman’s rank correlation coefficient = − 0.736) and lowest for EQ-5D in the non-TBI group (Spearman’s rank correlation coefficient = − 0.652). For both TBI and non-TBI groups, the explanatory power of EQ-5D + C was slightly higher than of EQ-5D (*R*^2^ = 0.56 vs. 0.53 for TBI; *R*^2^ = 0.47 vs. 0.45 for non-TBI). Absolute classification efficiency was higher for EQ-5D + C than for EQ-5D in both TBI groups, whereas relative classification efficiency was similar.

**Conclusions:**

Psychometric performance in general of both the EQ-5D and EQ-5D + C was better in TBI patients. Adding a cognitive bolt-on slightly improved the psychometric performance of the EQ-5D-3L.

## Background

Currently, the measurement of health-related quality of life (HRQoL) is standard practice in evaluating the impact of health interventions [1]. HRQoL instruments can be categorized as generic and disease-specific measures, where a particular subclass of instruments are preference-based or ‘utility’ measures, which can be used in economic evaluations [2].

One of the generic instruments that is widely implemented is the EQ-5D. The EQ-5D is a self-assessment instrument that consists of five items on the following dimensions: mobility, self-care, usual activities, pain/discomfort, and anxiety/depression and a visual analogue scale (VAS) [3]. The five dimensions can be scored using 3- or 5-level ordinal response options. One major advantage of the EQ-5D over other generic HRQoL measurement instruments is its brevity and subsequent low burden to fill out [4, 5]. However, a downside of the brevity may be that important information for HRQoL is not included in the EQ-5D dimensions, resulting in an instrument that may be unable to capture certain health effects and that is not sensitive for the measurement of HRQoL of all conditions [6, 7]. Moreover, the EQ-5D is known to measure mainly physical dimensions of health, and lacking information on social domains and sensitivity on psychological domains. Therefore, the EQ-5D is inconsistent in some populations [8]. A solution to increase coverage of the EQ-5D may be to add a dimension (a ‘bolt-on’) covering a specific health problem or dysfunction relevant to any particular condition or disease [9].

Over time, different bolt-on items to enrich the EQ-5D have been suggested. One of these bolt-on items is cognition [10]. Cognition can be operationalized with attributes like concentration, memory, intellectual competence, and coherence [10]. A cognition bolt-on can be motivated from theoretical reasons or from the pragmatic observation that cognition affecting diseases and conditions are common (dementia, Parkinson’s disease, injury, birth trauma, and congenital neurological conditions).

Even though many studies already use the cognition bolt-on in the measurement of HRQoL with the EQ-5D, limited evidence on the added value of the cognitive bolt-on in patient groups with cognitive impairments exists. Wolfs et al. [11] found that in a population of elderly patients with cognitive impairments, the addition of a cognitive dimension had no effect on the construct validity. Ophuis et al. (Working paper: Health-related quality of life in injury patients: the added value of extending the EQ-5D-3L with a cognitive dimension) found that adding a cognition dimension to the EQ-5D in a heterogeneous sample of injury patients, including patients with mostly mild traumatic brain injury (TBI), had a similar impact on EQ-VAS scores as any of the existing five EQ-5D dimensions. However, both studies were lacking a clear differentiation between the effects of adding a cognitive dimension to the EQ-5D in patients with and without suspected cognitive impairments. Therefore, this study can provide a more accurate insight in the additional value of a cognition dimension in the EQ-5D for TBI patients.

This study investigated the added value of a cognition dimension to the EQ-5D in a population of injury patients. The added value was analyzed in the form of the distributional benefit, the convergent validity, explanatory power, and classification efficiency of the EQ-5D and EQ-5D + C in a large sample of TBI and non-TBI patients who were admitted to the hospital due to their injury. We explored this by comparing ceiling and other distribution effects and convergent validity using the predictive association with the EQ-VAS. Also, we assessed the explanatory power using regression analyses, and classification efficiency using Shannon indices. Each of these aspects were determined for patients with and without TBI.

## Methods

### Research population and data collection

The Brabant Injury Outcome Surveillance (BIOS) study is a prospective cohort follow-up study of adult injury patients (18 years or older) who were admitted to one of the 10 participating hospitals through the emergency department (ED), and who survived until hospital discharge [12]. The inclusion of the study was conducted from 1 August 2015 until 30 November 2016. The exclusion criteria were the inability to understand the Dutch language, the absence of a permanent address of residence, and the suspected presence of a pathological fracture due to a malignancy or metastasis [12]. The study was approved by the Medical Ethics Committee of the Province of Brabant (METC code: NL50258.028.14).

The information on age, gender, nature, and severity of the injury were derived from the hospital registries. The severity of the injury was defined by the Injury Severity Score (ISS) [13]. The ISS is based on the square of the highest Abbreviated Injury Scales (AIS) of the three most severely injured body regions. The AIS describes the type, location, and severity of an injury [14]. Patients with an ISS of 16 and more are defined as severely injured. With the AIS score, it can be determined whether a patient has TBI, and what the severity level of the TBI is (mild, moderate, or severe). TBI was defined as mild with AIS head < 3, moderate with AIS head = 3, and severe with an AIS head > 3 [12]. Additional data were collected by a postal survey that patients received 1 month after injury [12]. The survey included the EQ-5D + C, the EQ-VAS, the highest level of education, and the existence of comorbidity.

### HRQoL data

The EQ-5D consists of five items on different dimensions of health (one item per dimension): mobility, self-care, usual activity, pain/discomfort, and anxiety/depression. The questions on the different dimensions are available in two versions: a three-level version and a five-level version. We used the three-level version (EQ-5D-3L). In the 3L version, the answer options to each question are ‘no problems,’ ‘some problems,’ and ‘extreme problems’/‘unable to perform.’ Data can be represented by means of a profile summarizing the respondents’ reported health problems defining the severity level (where 1 means no problems), e.g., ‘21332,’ and used in a descriptive way and, after summarizing response into an unweighted summary score, also referred to as ‘misery index’ (range 0–15) or a so-called 0–1 utility score. Furthermore, the respondents were asked to rate their health from 0 to 100 on a VAS scale, where 0 is the worst imaginable health state, and 100 is the best imaginable health state. The score that is provided in this question by the respondent is the EQ-VAS score.

In the BIOS study, an additional dimension was added to the EQ-5D questionnaire for cognition. This bolt-on should capture information on cognitive functioning, operationalized as concentration, memory, and IQ [10]. The cognition bolt-on consisted of one question, and was framed like the other dimension questions, with the same number of response options [3]. The instruction was (translated from the Dutch questionnaire): By placing a check mark in one box in each group below, please indicate which statement best describes your own state of health. The cognition bolt-on item was worded as follows: cognition (such as memory, concentration). The answer options were: I have no problems with my cognitive functioning; I have some problems with my cognitive functioning; I have severe problems with my cognitive functioning.

### Data analysis

The data analyses were performed with SPSS version 24. The responses of the patients to the question on the highest level of education were categorized in a variable with the values low, medium, and high education level. Comorbidity status was determined per patient as the number of pre-existing conditions. Respondents were included in the analyses if all questions of the EQ-5D including the cognition dimension and the EQ-VAS score were answered. The frequencies of the socio-demographics were determined with frequency analyses in SPSS. Furthermore, independent sample *t* tests were performed on the frequencies of the socio-demographics, comparing the group of TBI patients with the group of non-TBI patients. To determine whether there was a distributional effect in terms of ceiling effect, the proportion of perfect health profiles (11111 for EQ-5D and 111111 for EQ-5D + C) among all observed profiles was determined (the higher the share, the more ceiling).

The convergent validity of the EQ-5D and the EQ-5D + C was measured by determining the association between the EQ-VAS, the EQ-5D, and the EQ-5D + C, respectively. We first calculated the misery index for both the EQ-5D and the EQ-5D + C, consisting of the sum of the levels of the five dimensions (e.g., health profile 11111 had a misery index of 5), where the highest score 15 represents the poorest health. Next, Spearman’s rank correlation coefficients between the EQ-5D and EQ-5D + C misery indices and EQ-VAS were determined for TBI and non-TBI patients, after it was confirmed that the assumptions of the Spearman’s rank correlation were met [15].

In order to determine the explanatory power of EQ-5D and EQ-5D + C, we performed univariate and multivariable analyses using the EQ-VAS as dependent variable. With the univariate analyses we tested whether all dimensions of the EQ-5D(+ C) were related to the EQ-VAS. The levels ‘some problems’ and ‘extreme problems’ of all dimensions, including the cognitive dimension, were used to predict the EQ-VAS. The two severity levels were recoded into dummy variables, with the ‘no problems’ level as the reference category. With each of the dummy variables, the EQ-VAS score was then predicted. Thereafter, multivariable analyses were done, as the assumptions of the linear regression model were met, with different combinations of the dimensions of the EQ-5D and the cognitive dimension in the model. The combinations consisted of the original EQ-5D dimensions, the EQ-5D dimensions with the cognitive dimension, and all combinations of five out of the six dimensions. An additional model was tested for the EQ-5D and the EQ-5D + C for both groups with the backward deletion strategy.

To determine the classification efficiency of both the EQ-5D and the EQ-5D + C, the Shannon index (H’) and the Shannon Evenness index (J’) were determined [16]. These two indices provide information on the ability of the EQ-5D(+ C) to measure diversity in the population [17]. The Shannon index was calculated with the following formula: *H*′ = − ∑^*c*^_*i*=1_*p*_i_^2^log *p*_i_, where p_i_ is the proportion of people with a certain health profile, and *C* is the total number of possible health profiles. The higher the value of *H*′, the more information is captured by the EQ-5D or EQ-5D + C. The total number of possible health profiles is 3*3*3*3*3 = 243 for the EQ-5D, and 729 for the EQ-5D + C. The Shannon Evenness index was calculated with the formula: *J*′ = *H*′/*H*′_max_, where *H*′_max_ is ^2^logC and indicates the total number of possible health profiles. The Shannon Evenness index increases if the extra dimension is used to make more distinction between patients and flattens the distribution into more different health profiles [18]. According to Pielou [19], any assessment of *H*′ using a sample of the total ‘true’ population will lead to an underestimation of information captured, with a magnitude of (*C* − 1)/2*N*. This underestimation may be considerable when *C* is large such as in the EQ-5D, but especially EQ-5D + C. Therefore, we also calculated adjusted values of *H*′ and *J*′, taking this underestimation bias into account.

### Hypotheses

The following five hypotheses were formulated:

- The ceiling with the EQ-5D + C is less than with the EQ-5D;
- The convergent validity of the EQ-5D + C with the EQ-VAS is higher than the convergent validity of the EQ-5D with the EQ-VAS in the group of TBI patients;
- The explanatory power of the EQ-5D + C is higher compared to the EQ-5D in TBI patients due to specific cognitive symptoms after TBI;
- The explanatory power of the EQ-5D is higher in non-TBI patients than in TBI patients due to more heterogeneity in type and nature of injury;
- Absolute classification efficiency of the EQ-5D + C is higher compared to the EQ-5D in the group of TBI patients, while relative classification efficiency is similar.

## Results

### Study population

**Fig. 1:**
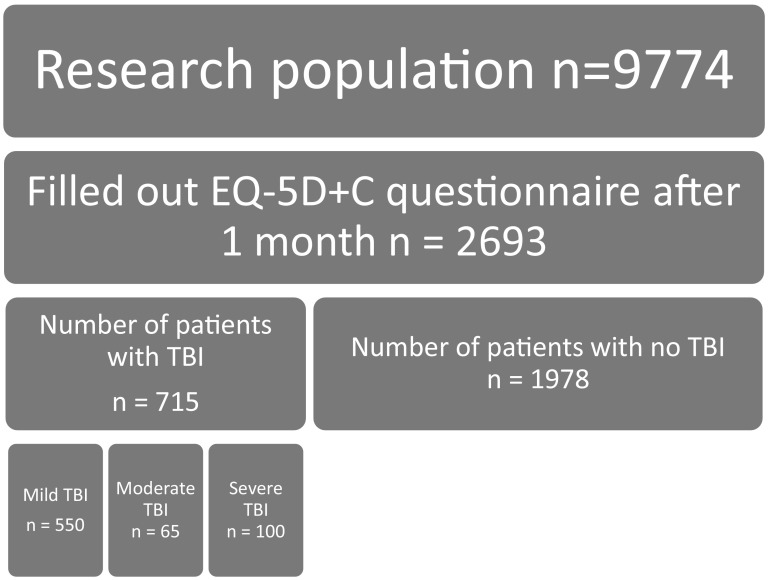
Patient flow chart

The flow chart of the selection of the study population can be found in Fig. 1. In total, 9774 people received a questionnaire 1 month after injury. Out of these 9774 people, 2693 (27.6%) filled out each item of the EQ-5D + C and EQ-VAS. Approximately, one in four was diagnosed with TBI (*n* = 715; 26.6%), where the majority of the TBI patients suffered from mild TBI (*n* = 550; 76.9%). The socio-demographics of the respondents are summarized in Table 1. The proportion of males was significantly higher for the group of TBI respondents (57.3%) than for the group of non-TBI respondents (48.4%) (*p* < 0.05). TBI patients reported problems most frequently on the pain dimension (70.1%), whereas the non-TBI patients reported problems most frequently on the usual activities dimension (87.3%). The average ISS score was significantly higher for the group of non-TBI respondents than for the group of TBI respondents (6.83 vs. 6.05, *p* < 0.05).

**Table 1:** Characteristics of research population

Demographics of research population	Research population	TBI	Non-TBI	*p* value
*n*	2693	715	1978	
Mean age (SD)	64.0 (18.3)	61.4 (18.6)	65.0 (18.1)	< 0.001*
Females	1326 (49.2%)	305 (42.7%)	1021 (51.6%)	< 0.001*
Education level				
Low	898 (33.3%)^a^	233 (32.6%)^b^	665 (33.6%)^c^	0.616
Medium	963 (35.8%)^a^	258 (36.1%)^b^	705 (35.6%)^c^	0.833
High	752 (27.9%)^a^	198 (27.7%)^b^	554 (28.0%)^c^	0.872
ISS scores				
1–3	609 (22.6%)^d^	358 (50.1%)	251 (12.7%)^d^	< 0.001*
4–8	921 (34.2%)^d^	169 (23.6%)	752 (38.0%)^d^	< 0.001*
9–15	1005 (37.3%)^d^	125 (17.5%)	880 (44.5%)^d^	< 0.001*
16+	141 (5.2%)^d^	63 (8.8%)	78 (3.9%)^d^	< 0.001*
Severity of TBI				
Mild	–	550 (76.9%)	–	–
Moderate	–	65 (9.1%)	–	–
Severe	–	100 (14.0%)	–	–
Comorbidity				
*n* pre-existing conditions				
0	1061 (39.4%)	306 (42.8%)	755 (38.2%)	0.032*
1	724 (26.9%)	194 (27.1%)	530 (26.8%)	0.861
2	441 (16.4%)	111 (15.5%)	330 (16.7%)	0.473
3+	467 (17.4%)	104 (14.5%)	363 (18.5%)	0.016*

*SD* standard deviation

*Significant at 5% level

^a^80 missing values

^b^26 missing values

^c^54 missing values

^d^17 missing values

### Frequency of unique health states and ceiling effects

Of the 2693 respondents, 214 people (7.9%) reported a perfect health state on the EQ-5D, and 197 (7.3%) on the EQ-5D + C. In the group of TBI patients, there were 129 (18.0%) with perfect health on the EQ-5D, and 117 (16.4%) with perfect health on the EQ-5D + C. In the non-TBI group, the numbers were 85 (4.3%) for the EQ-5D, and 80 (4.0%) for the EQ-5D + C.

### Convergent validity

Table 2 shows that convergent validity was highest for TBI group (EQ-5D + C: Spearman’s rank correlation coefficient = − 0.736; EQ-5D: Spearman’s rank correlation coefficient = − 0.719) and lowest in the non-TBI group (EQ-5D + C: Spearman’s rank correlation coefficient = − 0.665; EQ-5D: Spearman’s rank correlation coefficient = − 0.652). Note that health increases with a lower score in EQ-5D misery index and a higher score in EQ-VAS.

**Table 2:** Spearman’s rank correlation sum score EQ-5D and EQ-VAS and sum score EQ-5D + C and EQ-VAS for TBI and non-TBI patients

	Combination	Spearman’s rank correlation
Research population	EQ-5D – EQ-VAS	− 0.673
	EQ-5D + C – EQ-VAS	− 0.690
TBI	EQ-5D – EQ-VAS	− 0.719
	EQ-5D + C – EQ-VAS	− 0.736
Non-TBI	EQ-5D – EQ-VAS	− 0.652
	EQ-5D + C – EQ-VAS	− 0.665

### Explanatory power

The univariate regression analyses of the EQ-5D + C dimensions showed that all dimensions were significantly associated with the EQ-VAS score in both the group of TBI patients and the group of non-TBI patients (Appendix Table 5). The observed directions of the dimension effects were in all cases as expected (more problems, negative coefficient), except for the level 2 effect of usual activities in the non-TBI group. Also, the magnitude of the effects (level 3 larger than level 2) generally conformed to the expectations. The explained variance of the EQ-VAS was in the TBI group highest for usual activity level 3 (24.3%). In the non-TBI group, the explained variance of the EQ-VAS was highest for self-care level 3 and usual activities level 3 (both 20.4%). The explained variance of the EQ-VAS by the cognition dimension was higher in the TBI group than in the non-TBI group for level 2, as expected, but lower for level 3, which was unexpected (8.0% for level 2 and 9.9% for level 3 vs. 4.5% for level 2 and 12.0% for level 3).

**Fig. 2:**
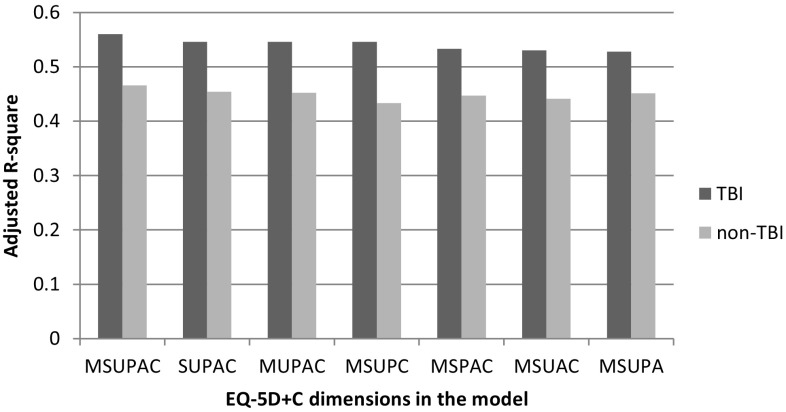
Adjusted *R*^2^ per EQ-5D dimensions included for TBI and non-TBI patients for the EQ-VAS. *M* mobility, *S* self-care, *U* usual activities, *P* pain/discomfort, *A* anxiety/depression, *C* cognition

The results of the multivariable regression analyses of the group of TBI patients and non-TBI patients are displayed in Table 3. Generally, 55% of the variance of the EQ-VAS could be explained in TBI patients, and 45% in non-TBI patients. Adding the cognition dimension to the EQ-5D model resulted in an increase in explanatory power from 52.8 to 56.0% for the TBI group, and from 45.1 to 46.6% for the non-TBI group. A mutual comparison of explanatory power of all five different selections of five dimensions from six dimensions resulted in similar explained variances, ranging from 52.8 to 54.6% in TBI patients and 43.3 to 45.4% in non-TBI patients (see also Fig. 2). The models with additional explanatory variables, resulting from the backward deletion strategy, did provide little additional explanatory power in all cases, except for the EQ-5D + C for TBI patients, where it provided no extra power. The model for the EQ-5D for TBI patients provided an additional explanatory power of 0.9% when the variable ‘Number of TBIs’ was added. For the group of non-TBI patients, an additional explanatory power of 1.1% was found for the EQ-5D + C when the ISS and the number of comorbidities were added, and an additional explanatory power of 1.5% in the EQ-5D when the same two variables were added.

**Table 3:** Explanatory power of multivariable models for the EQ-VAS that include any combination of the EQ-5D and cognition dimensions for TBI and non-TBI patients

Combination of EQ-5D + C dimensions	Adjusted *R*^2^	*F* value	*P* value
Research population			
MO, SC, UA, PD, AD	0.480	249.1	< 0.001*
MO, SC, UA, PD, AD, CO	0.499	224.5	< 0.001*
MO, UA, PD, AD, CO	0.486	255.4	< 0.001*
MO, SC, PD, AD, CO	0.477	246.8	< 0.001*
MO, SC, UA, AD, CO	0.472	241.7	< 0.001*
MO, SC, UA PD, CO	0.471	241.2	< 0.001*
SC, UA, PD, AD, CO	0.487	256.4	< 0.001*
TBI patients			
MO, SC, UA, PD, AD	0.528	80.9	< 0.001*
MO, SC, UA, PD, AD, CO	0.560	76.6	< 0.001*
MO, UA, PD, AD, CO	0.546	86.7	< 0.001*
MO, SC, PD, AD, CO	0.533	82.3	< 0.001*
MO, SC, UA, AD, CO	0.530	81.5	< 0.001*
MO, SC, UA PD, CO	0.546	86.8	< 0.001*
SC, UA, PD, AD, CO	0.546	87.0	< 0.001*
Non-TBI patients			
MO, SC, UA, PD, AD	0.451	163.7	< 0.001*
MO, SC, UA, PD, AD, CO	0.466	145.0	< 0.001*
MO, UA, PD, AD, CO	0.452	164.3	< 0.001*
MO, SC, PD, AD, CO	0.447	161.1	< 0.001*
MO, SC, UA, AD, CO	0.441	157.2	< 0.001*
MO, SC, UA PD, CO	0.433	151.9	< 0.001*
SC, UA, PD, AD, CO	0.454	165.7	< 0.001*

*MO* mobility, *SC* self-care, *UA* usual activities, *PD* pain/discomfort, *AD* anxiety/depression, *CO* cognition

*Significant at 5% level

### Classification efficiency—Shannon’s indices

The number of different health profiles in the TBI group was considerably lower compared to the non-TBI group. In the group of TBI patients, 87 out of 243 (35.8%) possible EQ-5D profiles were used, and 153 out of 729 (21.0%) possible EQ-5D + C profiles were reported. In the group of non-TBI patients, 127 out of 243 (52.3%) possible EQ-5D profiles and 232 out of 729 (31.8%) possible EQ-5D + C profiles were reported. This was reflected in the Shannon index and the Shannon Evenness index, which were 5.08 (Shannon index) and 0.64 (Shannon Evenness index) for the EQ-5D and 5.88 (Shannon index) and 0.62 (Shannon Evenness index) for the EQ-5D + C, respectively, for the TBI group, while for the non-TBI group the results were 5.58 and 0.70 for the EQ-5D, and 6.38 and 0.67 for the EQ-5D + C (Table 4). Apart from the non-TBI group being more heterogeneous, this indicates that generally more information is captured by the EQ-5D + C compared to the EQ-5D. The Shannon Evenness index was higher for the EQ-5D than for the EQ-5D + C (*J*′ = 0.64 vs. *J*′ = 0.62 for TBI, and *J*′ = 0.70 vs. *J*′ = 0.67 for non-TBI), but were similar after adjusting for underestimation bias (*J*′_adj_ = 0.66 vs. *J*′_adj_ = 0.67 for TBI, and *J*′_adj_ = 0.71 vs. *J*′_adj_ = 0.69 for non-TBI). Apparently, more information is captured but the observed gain in discrimination in both groups is relatively low in view of the increase of classification options (adding cognition increases the options with 729 − 243 = 585 profiles).

**Table 4:** Shannon index (H′) and Shannon Evenness index (J′) of EQ-5D and EQ-5D + C for TBI and non-TBI patients

Health profiles	H′	Adjusted H′	J′	Adjusted J′
TBI				
EQ-5D	5.08	5.25	0.64	0.66
EQ-5D + C	5.88	6.39	0.62	0.67
Non-TBI				
EQ-5D	5.58	5.64	0.70	0.71
EQ-5D + C	6.38	6.56	0.67	0.69

## Discussion

### Main findings

This study addressed the potential gain of adding a cognitive dimension to the EQ-5D measure, in a large group of injury patients where this gain could be relevant in the subgroup of TBI patients. Genuinely it slightly improved ceiling effects and improved the ranking of patients if a self-rated VAS was used as reference. As expected, this effect was larger in TBI patients. However, the increase in explanatory power was rather small (3.2% if we focus on TBI). Furthermore, the added value in terms of explanatory power of the cognition dimension in the non-TBI population was close to zero, as expected. Discriminatory potential (informativity) was higher for the EQ-5D + C than for the EQ-5D in both groups, whereas relative informativity was similar.

### Comparison to previous studies

The addition of a cognition dimension to the EQ-5D has been studied before, but not specifically in a population with TBI. Ophuis et al. (working paper) looked into the effect in a small group of TBI patients, but a clinical classification of severity of the TBI was lacking. Moreover, in the study by Ophuis et al. (working paper) HRQoL was assessed 2.5 months after ED treatment, whereas in this study the EQ-5D(+ C) was assessed 1 month after ED treatment, and as a result we expect that less patients fully recovered from their injuries. Despite these differences, the findings of the study by Ophuis et al. (working paper) were similar to our findings: compared to EQ-5D the increase in explanatory power of the EQ-5D + C is small (< 3%).

Findings of other studies on the cognition dimension are more difficult to compare. Krabbe et al. [10] looked into the effect of adding a cognition dimension to the EQ-5D; however, in this study the content validity was tested, as well as the impact on utilities. The study by Jelsma and Maart [7] looked into the effect of adding several dimensions, including a concentration dimension, to the EQ-5D. Concentration is considered a part of the cognition dimension. The study by Jelsma and Maart [7] found that the addition of a concentration dimension increased the explanatory power of the model (52–55% when concentration, energy, body appearance, sleep, and sexual activity were added). However, they did not report the increase in explanatory value for each extra dimension separately.

Convergent validity of the EQ-5D has been studied by Golicki et al. [20]. In this study, the convergent validity was determined for each dimension of the EQ-5D with the EQ-VAS, with the Spearman rank correlation coefficients ranging from − 0.43 for pain/discomfort to − 0.75 for usual activities [20]. These correlation coefficients are in the same order of magnitude compared to those we found for the EQ-5D and EQ-5D + C.

Previous studies on the classification efficiency of the EQ-5D found varying values for the Shannon index and the Shannon Evenness index of the EQ-5D. Janssen, Birnie, and Bonsel [21] found a Shannon index of 6.37 and a Shannon Evenness index of 0.80 in a general population sample from the US. Polinder et al. [22] reported a Shannon index for the EQ-5D of 2.71 and a Shannon Evenness index of 0.53 in an injury population. For skull and brain injury patients, this study reported a Shannon index of 2.55 for the EQ-5D and a Shannon Evenness index of 0.49 [22]. The Shannon index that was found in our injury population is higher (5.08 for TBI and 5.58 for non-TBI). This can be explained by the fact that the measurement of HRQoL in our study was conducted 1 month after presentation at the ED, versus 2 years after injury. Therefore, it is likely that there was a larger variation of health profiles in our study than in the study by Polinder et al. [22], resulting in a higher Shannon index and Shannon Evenness index. It should be taken into account that the ceiling effect is also part of the formula to determine the classification efficiency. However, in calculating the classification efficiency it is not relevant whether a high frequency of one health profile is in the perfect health profile (11111) or in another health profile. Therefore, if a health profile is overrepresented, this will result in a smaller classification efficiency.

### Strengths and limitations

This study had several strengths and limitations. One of the strengths was that diagnosis and severity of TBI was registered. A limitation of this study was the timing of the HRQoL assessment. A study by Schretlen and Shapiro [23] found that cognitive functioning after mild TBI restores within 1–3 months. Since the majority of patients in the TBI group had mild TBI, patients may have already recovered 1 month after presentation at the ED. The BIOS study also contains data on a 1-week measurement; however, the number of respondents at this point is rather small. Secondly, the group of non-TBI patients had on average a significantly higher ISS score, indicating that this group of patients sustained more severe injuries compared to the group of TBI patients. This is reflected by the much higher proportion of patients in the TBI group who reported perfect health. The EQ-5D was found to suffer from ceiling effect, meaning that health states close to good health cannot be distinguished well [18, 24]. Therefore, the ceiling effect was larger in the TBI group than in the non-TBI group. A question that arises is whether the ceiling effect in the TBI group (18.0% for EQ-5D and 16.4% for EQ-5D + C) would have been reduced when the EQ-5D-5L or EQ-5D-5L + C was used, as is suggested by Janssen et al. [18]. Respondents with minor complaints on one of the dimensions will choose ‘no problems’ in the EQ-5D-3L, but are likely to choose ‘slight problems’ in the EQ-5D-5L.

For future research, we recommend to assess the added value of the cognitive bolt-on for the EQ-5D-5L and to include more patients with moderate or severe TBI to enable a comparison between the severity levels of TBI. Furthermore, we recommend to develop guidelines to determine what a meaningful difference, in a statistical sense, of a bolt-on is.

## Conclusion

In conclusion, the addition of a cognition dimension to the EQ-5D improves the convergent validity, explanatory power, and classification efficiency of the EQ-5D. However, improvements are rather small. The effect on the convergent validity and the explanatory power of the EQ-5D is larger in the group of TBI patients than in the group of non-TBI patients, suggesting that the additional value of a cognition dimension in the EQ-5D is especially relevant in a patient group with (expected) problems with cognition.

## Appendix

**Table 5:** Univariate analyses of EQ-VAS explained by the EQ-5D and cognition dimensions for TBI and non-TBI patients

EQ-5D dimension	*R* ^2^	Unstandardized B (95% CI)	*p* value
TBI patients			
Mobility level 2	0.116	− 13.4 (− 16.1 to − 10.7)	< 0.001*
Mobility level 3	0.109	− 33.3 (− 40.3 to − 26.3)	< 0.001*
Self-care level 2	0.076	− 12.1 (− 15.3 to − 9.0)	< 0.001*
Self-care level 3	0.156	− 28.3 (− 33.2 to − 23.5)	< 0.001*
Usual activities level 2	0.009	− 3.7 (− 6.6 to − 0.9)	0.005*
Usual activities level 3	0.243	− 24.2 (− 27.3 to − 21.0)	< 0.001*
Pain/discomfort level 2	0.038	− 7.8 (− 10.8 to − 4.9)	< 0.001*
Pain/discomfort level 3	0.131	− 26.3 (− 31.3 to 21.3)	< 0.001*
Anxiety/depression level 2	0.123	− 15.4 (− 18.4 to − 12.4)	< 0.001*
Anxiety/depression level 3	0.062	− 25.5 (− 32.8 to − 18.2)	< 0.001*
Cognition level 2	0.080	− 11.7 (− 14.5 to − 8.8)	< 0.001*
Cognition level 3	0.099	− 29.7 (− 36.3 to − 23.1)	< 0.001*
Non-TBI patients			
Mobility level 2	0.005	− 2.8 (− 4.6 to − 1.1)	0.002*
Mobility level 3	0.114	− 20.1 (− 22.6 to − 17.7)	< 0.001*
Self-care level 2	0.002	− 1.8 (− 3.5 to − 0.1)	0.043*
Self-care level 3	0.204	− 23.6 (− 25.6 to − 21.5)	< 0.001*
Usual activities level 2	0.050	8.8 (7.1 to 10.5)	< 0.001*
Usual activities level 3	0.204	− 18.0 (− 19.6 to − 16.5)	< 0.001*
Pain/discomfort level 2	0.003	− 2.4 (− 4.3 to − 0.5)	0.014*
Pain/discomfort level 3	0.088	− 19.8 (− 22.6 to − 17.0)	< 0.001*
Anxiety/depression level 2	0.095	− 13.3 (− 15.1 to − 11.5)	< 0.001*
Anxiety/depression level 3	0.102	− 28.4 (− 32.1 to − 24.7)	< 0.001*
Cognition level 2	0.045	− 9.9 (− 11.9 to − 7.9)	< 0.001*
Cognition level 3	0.120	− 26.5 (− 29.7 to − 23.4)	< 0.001*

*95% CI* = 95% confidence interval

*Significant at 5% level

## References

[1] Gabbe BJ, Simpson PM, Cameron PA, Ponsford J, Lyons RA, Collie A (2017). Long-term health status and trajectories of seriously injured patients: A population-based longitudinal study. PLoS Medicine.

[2] Patrick DL, Deyo RA (1989). Generic and disease-specific measures in assessing health status and quality of life. Medical Care.

[3] Brooks R (1996). EuroQol: The current state of play. Health Policy.

[4] Linde L, Sorensen J, Ostergaard M, Horslev-Petersen K, Hetland ML (2008). Health-related quality of life: Validity, reliability, and responsiveness of SF-36, 15D, EQ-5D [corrected] RAQoL, and HAQ in patients with rheumatoid arthritis. Journal of Rheumatology.

[5] Johnson JA, Coons SJ, Ergo A, Szava-Kovats G (1998). Valuation of EuroQOL (EQ-5D) health states in an adult US sample. Pharmacoeconomics.

[6] Lin FJ, Longworth L, Pickard AS (2013). Evaluation of content on EQ-5D as compared to disease-specific utility measures. Quality of Life Research.

[7] Jelsma J, Maart S (2015). Should additional domains be added to the EQ-5D health-related quality of life instrument for community-based studies? An analytical descriptive study. Population Health Metrics.

[8] Finch AP, Brazier JE, Mukuria C (2018). What is the evidence for the performance of generic preference-based measures? A systematic overview of reviews. The European Journal of Health Economics.

[9] Yang Y, Rowen D, Brazier J, Tsuchiya A, Young T, Longworth L (2015). An exploratory study to test the impact on three “bolt-on” items to the EQ-5D. Value Health.

[10] Krabbe PF, Stouthard ME, Essink-Bot ML, Bonsel GJ (1999). The effect of adding a cognitive dimension to the EuroQol multiattribute health-status classification system. Journal of Clinical Epidemiology.

[11] Wolfs CA, Dirksen CD, Kessels A, Willems DC, Verhey FR, Severens JL (2007). Performance of the EQ-5D and the EQ-5D + C in elderly patients with cognitive impairments. Health Qual Life Outcomes.

[12] de Jongh MA, Kruithof N, Gosens T, van de Ree CL, de Munter L, Brouwers L (2017). Prevalence, recovery patterns and predictors of quality of life and costs after non-fatal injury: The Brabant Injury Outcome Surveillance (BIOS) study. Injury Prevention.

[13] Baker SP, O’Neill B, Haddon W, Long WB (1974). The injury severity score: A method for describing patients with multiple injuries and evaluating emergency care. The Journal of Trauma.

[14] Gennarelli TA, Wodzin E (2006). AIS 2005: A contemporary injury scale. Injury.

[15] van der Molen T, Willemse BW, Schokker S, ten Hacken NH, Postma DS, Juniper EF (2003). Development, validity and responsiveness of the Clinical COPD Questionnaire. Health and Quality of Life Outcomes.

[16] Shannon CE (1997). The mathematical theory of communication 1963. MD Computing.

[17] Pickard AS, De Leon MC, Kohlmann T, Cella D, Rosenbloom S (2007). Psychometric comparison of the standard EQ-5D to a 5 level version in cancer patients. Medical Care.

[18] Janssen MF, Pickard AS, Golicki D, Gudex C, Niewada M (2013). Measurement properties of the EQ-5D-5L compared to the EQ-5D-3L across eight patient groups: A multi-country study. Quality of Life Research.

[19] Pielou EC (1966). Shannon’s Formula as a measure of specific diversity: Its use and misuse. The American Naturalist.

[20] Golicki D, Niewada M, Buczek J, Karlinska A, Kobayashi A, Janssen MF (2015). Validity of EQ-5D-5L in stroke. Quality of Life Research.

[21] Bas Janssen MF, Birnie E, Bonsel GJ (2007). Evaluating the discriminatory power of EQ-5D, HUI2 and HUI3 in a US general population survey using Shannon’s indices. Quality of Life Research.

[22] Polinder S, Haagsma JA, Bonsel G, Essink-Bot ML, Toet H, van Beeck EF (2010). The measurement of long-term health-related quality of life after injury: Comparison of EQ-5D and the health utilities index. Injury Prevention.

[23] Schretlen DJ, Shapiro AM (2003). A quantitative review of the effects of traumatic brain injury on cognitive functioning. International Review of Psychiatry.

[24] Brazier J, Roberts J, Tsuchiya A, Busschbach J (2004). A comparison of the EQ-5D and SF-6D across seven patient groups. Health Economics.

